# Exploring the entropic nature of political polarization through its formulation as a isolated thermodynamic system

**DOI:** 10.1038/s41598-023-31585-w

**Published:** 2023-03-17

**Authors:** Alexander V. Mantzaris, George-Rafael Domenikos

**Affiliations:** 1 Department of Statistics and Data Science, University of Central Florida, Orlando, 32816 USA; 2 Laboratory of Applied Thermodynamics, Thermal Engineering Sector, School of Mechanical Engineering, National Technical University of Athens, Heroon Polytechniou 9, Zografou, 15780 Athens, Greece

**Keywords:** Complex networks, Computational science

## Abstract

Political polarization has become an alarming trend observed in various countries. In the effort to produce more consistent simulations of the process, insights from the foundations of physics are adopted. The work presented here looks at a simple model of political polarization amongst agents which influence their immediate locality and how a entropy trace of the political discourse can be produced. From this model an isolated system representation can be formulated in respect to the changes in the entropy values across all variables of the system over simulation time. It is shown that a constant entropy value for the system can be calculated so that as the agents coalesce their opinions, the entropy trace in regards to political engagements decreases as the entropy value across non-political engagements increase. This relies upon an intrinsic constraint upon agents imposing a fixed number of activities per time point. As a result the simulation respects the second law of thermodynamics and provides insight into political polarization as a basin of entropy within an isolated system without making assumptions about external activities.

## Introduction

*Social physics* has laid out an interesting set of goals where natural sciences and social sciences would come together in order to help model human based systems^1,2^ (that the “verbal reasoning” in the humanities would be assisted by science and technology). From a recent review in the field of social physics^3^, it can be appreciated that the overlap between physics and a myriad of social phenomena has allowed researchers to understand how certain processes are governed. This involves investigating the underlying dynamics between the granular elements, the generative processes, how to visualize complex systems, and other aspects of dynamics around human centered ad-hoc interactivities. The work of^3–5^ provide a thorough review of much of the approaches in modeling social activities from a physics perspective highlighting the techniques adopted from physics which have been used successfully in studying datasets arising from human activities. Applications demonstrate new approaches to the statistical physics of crime^6^, climate change dilemmas^7^, social media polarization^8^, and far reaching topics including even the entropy and complexity of the evolution of memes^9^ are explored. Ideas along this line can even be traced back earlier publications^10^.

One of the most important principles in physics is that of the second law of thermodynamics which states that the the entropy of the system cannot decrease over time^11^. There is notable previous work incorporating this principle in general complex systems such as^12^ (global climate)^13,14^, (ecosystems)^15^, (entropy pertaining to wealth), and^16^ which looks at entropy in the field of economics. In general there is the question, “does the second law of thermodynamics apply to social systems or not?”^17^. Bailey^18^ discusses how the 2nd law of thermodynamics is prevalent in all living and nonliving entities regardless of the layers of complexity they rely upon. That regardless of the perspective of the system viewed this law will be acting^19^.

In the work of^20^ the Schelling model of segregation^21,22^ has its entropy trace along the simulation produced showing a decrease with the increased agent homogeneity, and in^23^ it is shown how with a dual dynamic operating on an income variable that the overall extended Schelling system can display an increasing entropy trace and respect the second law of thermodynamics. The motivation for the inclusion of a monetary variable as influencing the residential movements was inspiration from the work of^24^ that observed this from real world data collected. The second law of thermodynamics, also referred to as the *arrow of time*^25^, provides a direction (gradient) for which the combinatorical nature of a system can be expected to move in. Such work helps establish that social system models can be designed to respect the arrow of time since a decrease produces the Gibbs paradox due to an incomplete system definition^26–28^. There are other notable investigations into the social physics of the Schelling model such as^29^ which looks at the phase transitions, and the work of^30,31^ that are examples of research which makes the connection between the Schelling model and the Ising model of ferromagnetism.

From these approaches mentioned, and those discussed in the review articles, the system variables are not modeled as a completely isolated system. In the fields of physics and mechanical engineering it is common for researchers to devise a isolated system where the progression of the simulations result in a constant value of energy as the variables fluctuate according to the underlying dynamics^26,32,33^. There are many benefits produced by completely defining the system in isolation so that the exchange of entropy contributions between the system variables over simulation time can be examined. Adopting this paradigm into the social physics field can potentially allow deeper insight to be gained and produce a deeper understanding of the results due to constraints on the system variables.

In^23^ the extension to the Schelling model has a monetary dynamic associated with every move an agent makes on the grid that affects an income variable. This variable and dynamic does have the effect where the overall system entropy can be seen to increase due to the identity entropy decrease that is matched and overcome by the increase in the monetary entropy component. Such a new variable introduction is a plausible proposition given evidence from^24,34^, but it can be considered as a modeler selected incorporation since other variables may exist that can provide a similar dynamic which alleviates the physical violation. As an alternative we can take into account the natural constraints that the system agents can be expected to be bounded by in regards to the activities of concern. There is also a wide range of constraints that can be introduced but the most fundamental one (even more than the monetary component), is that of time. The agents can be thought of allocating their constrained time in engaging in a finite set of activities over simulation iterations. This constraint helps in defining the system which will be modeled as an ’isolated system’ with constant entropy.

These principles are integrated into a simple model of political polarization^35,36^ where agents reside in fixed positions within a lattice. Each agent is considered to have a single value for their political affiliation^37^ and strength between two choices^38^. Over simulation time the agents have these affiliation values influenced by their immediate neighbors^39^ which then can affect their political positions^40,41^. The agents are not able to change grid positions and begin with randomized affiliations and are not engaged in political discourse/influence when there is no ideological disparity^42,43^ with their locality (^44,45^). As will be shown in the Methodology section the entropy for the distribution of the agent variables in respect to the political affiliation and influence can be calculated using Shannon entropy. The entropy for the system in regards to the political activity can then be produced allowing for the entropy trace to be examined. When the agents’ activity for political actions decreases, the time constrained number of activities increases the agent activity in non-political engagements (peripheral). With this approach the agents are engaged in a constant total number of actions which changes the allocations between political and non-political (peripheral) activities. The peripheral activities introduce a component of uncertainty since the array of different actions (eg. walking, reading etc) are unknown to the modeler and provide a value of entropy as a result. The agents distribute their total activity number between political and peripheral actions for the total system entropy which is shown to be constant (over all time points). This allows the examination of the social model under this constraint to be an isolated system. It should be noted that this model is devised to explore the modeling paradigm for which a social system can be modeled in order to control for the entropy of the system rather than fit it to a real non-isolated system. The agent dynamics do not operate directly on the value of the system entropy or its components, and are agnostic to the values. The entropy trace is calculated in order to describe the system state for the modeler. A required assumption for the system described is for it to be isolated and bounded. By defining the entropy of the system a connection between its behaviors and the second law of thermodynamics is made. This gives the opportunity to acquire greater insight to the expected behaviors of such systems, and also lays the ground work for defining and studying the rest of the thermodynamic variables in social systems like the equivalent of the energy. This approach of using the entropy as the core variable to describe the behaviors of systems has also found applications in pure physics, like in the works of Bekenstein^46^ and Susskind^47^ considering the holographic principle, utilizing among others the maximum entropy of systems.

The Results section begins by showing how the model dynamics drive the agents towards greater political ideological homogeneity, and how this produces a decreasing entropy trajectory along the variable of the political actions. The analysis of the non-political actions (peripheral actions) shows a corresponding increasing entropy trajectory. Together it is shown how this inverse relationship produces a constant entropy value for the system where the simulation and theoretical calculations agree on the constant value. This inverse relationship rooted in a constraint, is reminiscent of the fundamental relationship $T \propto PV$ which is explored in the work of^48^ that investigates demographic distributions based upon this component of thermodynamics. The different values on the number of total actions, that can be taken by agents, is considered to be analogous to the temperature, the political actions to the pressure, and the peripheral actions to the volume.

## Methodology

The simple model of ideological exchange proposed here assumes that there is a square lattice (an $N\times N$ grid) where in each cell of the grid an agent resides which cannot change its position. Every agent has a ’contained’ political affiliation which is initially sampled uniformly from $\mathcal {U}[-Cmax,Cmax] \in \mathbb {Z}$ ($C_{max}=4$^49^), and these values are referenced from a matrix *C*. Agents can influence the contained political affiliation values in those agents found in their immediate adjacency (similar to the locality of the Schelling model and the Ising model of ferromagnetism^50^). There is a dynamic which governs how the values of an agent’s surrounding neighbors affect its own value. Given the iterations of the simulation, time steps, these values can continue to change as the dynamics are repeatedly applied. At the base of the model is the dynamic of how agents, which contain values $C_{i,j}$ (representing political affiliation strengths), change this contained political stance over time as agents influence each other from their immediate adjacency. Utilizing the matrix *C*, 3 more matrices are defined based upon the values in *C*; *M*, *I*, and *E*.

A matrix *M*, holding binary numbers, is defined to represent the result of how each agent, (*i*, *j*), will vote based on the values in *C*. The matrix *I* is defined to provide the aggregate value of all the voting decisions taken by agents in adjacent cells with values stored in *M*, excluding itself. These values in *I*, $I_{i,j}$, can be thought of as a bias quantity which an agent at position (*i*, *j*) is subjected to when there is not an equal number of neighbors voting for each side of the political spectrum. The matrix, *E*, holds values indicating whether an agent at position (*i*, *j*) will be active engaging in political discourse in an attempt to influence agents in the immediate neighborhood when there lacks an agreement (disparity) in that locality for the voting direction. In a state where each agent agrees on the voting directions stored in *M*, there are no political actions/engagements being taken by agents and each $E_{i,j}$ will be zero. Simulations are run with iteration numbers denoted by *t* and the matrix values change as the agents affect the values contained in *C* of their neighborhood. Table 1 provides a listing of the matrices proposed and a succinct description of their purpose.

**Table 1:** The matrices proposed and a description of their use.

Matrix	Description
$C_{i,j}$	Holds the values of an agent’s contained political affiliation $[-Cmax,\ldots ,Cmax]$.
$M_{i,j}$	The value for an agent’s voting decision in [0, 1] based upon $C_{i,j}$
$I_{i,j}$	A value in $[-8,8]$ representing whether the neighborhood of (*i*, *j*) has an ideological bias which an agent placed at (*i*, *j*) is subjected to; unbalanced voting directions from agents in adjacent cells
$E_{i,j}$	A binary value [0, 1] that is 1 when an agent is engaged in political discourse since agents in adjacent cells do not agree on a common political direction to vote on

$C_{i,j}$ for each simulation time point *t* ($t \in [1,\ldots ,T]$), $C_{i,j,t}$ is defined to be

1 $$\begin{aligned} \begin{aligned} C_{i,j,t}&= \sum _{m=i-1}^{i+1} \sum _{n=j-1}^{j+1} C_{i,j,t-1}+&{\left\{ \begin{array}{ll} +1\quad \,if\, C_{m,n,t-1}>0 \wedge C_{i,j,t-1}<C_{max} \wedge (i\ne m \wedge j\ne n)\\ -1\quad \,if\,C_{m,n,t-1}<0 \wedge C_{i,j,t-1}>-C_{max} \wedge (i\ne m \wedge j\ne n) \\ 0\quad \,if\,C_{m,n,t-1}=0 \wedge (i\ne m \wedge j\ne n) \\ 0\quad \,if (m<1) \vee (n<1) \vee (m>N) \vee (n>N) \end{array}\right. } . \end{aligned} \end{aligned}$$

In the situation that an agent is in the center of the ideological spectrum, ie $C_{i,j,t-1}=0$ then in the next iteration their influence will be inconsequential.

An agent manifests its $C_{i,j}$ value projected onto a vote aligning with the side of the political spectrum they lean towards. These values are held in a matrix *M*, $M \in \mathbb {R}^{N\times N}$, where $M_{i,j}$ values are given by:

2 $$\begin{aligned} M_{i,j,t} = {\left\{ \begin{array}{ll} 1\quad \,if\, C_{i,j,t}>0\\ 0\quad \,if\,C_{i,j,t}<0 \end{array}\right. }, \end{aligned}$$

where $M_{i,j,t} \in [0,1]$. If $C_{i,j,t}=0$ then $M_{i,j,t}=M_{i,j,t-1}$; the agent does not change the voting direction from the previous timestep.

Another variable, $I_{i,j}$ ($I \in \mathbb {R}^{N\times N}$), is introduced that represents the cumulative effect of the influencing neighborhood upon each agent and is necessary as it describes the ideological ’slant’/’bias’ of the locality experienced by an agent at each time step. In combination with the *C* matrix this will determine how the agent will be affected in the following time step without the requirement to know the entirety of the grid. It is found via:

3 $$\begin{aligned} I_{i,j,t} = \sum _{m=i-1}^{i+1} \sum _{n=j-1}^{j+1} {\left\{ \begin{array}{ll} +1\quad \,if\, M_{m,n,t}=1 \wedge (i\ne m \wedge j\ne n)\\ -1\quad \,if\,M_{m,n,t}=0 \wedge (i\ne m \wedge j\ne n) \\ 0\quad \,if\,C_{m,n,t}=0 \wedge (i\ne m \wedge j\ne n) \\ 0\quad \,if\, (m<1) \vee (n<1) \vee (m>N) \vee (n>N) \end{array}\right. } . \end{aligned}$$

Each entry can take the values $I_{i,j,t} \in [-8,\ldots ,8], I_{i,j,t} \in \mathbb {Z}$. In an idealistic societal state it could be aspired to have $I_{i,j,t} = 0$, as every neighbor is then exposed to a balanced set of opinions/leanings, but when that is not the case $\Vert I_{i,j,t}\Vert > 0$. These values can be found for the whole grid providing a measurement for the overall ideological bias the agents are exposed to regardless of their opinion against/for the bias. Such biases can be considered to be a potential threat as individuals are not exposed to a balanced set of ideas creating localized rifts which can therefore lead to polarization.

The entropy of this system can be calculated given macrostates of *C*, *M* and *I*. The macrostate values for *C* will be, $C_{v_{C}} = \sum _{i=1}^N\sum _{j=1}^N \delta _{C_{i,j},v_C}$ with $v_C\in [-Cmax,Cmax]$. This produces a set of values to explore the distribution for different arrangements of ideological positions of each $C_{i,j}$.The macrostate values for *M* will be, $M_{v_{M}} = \sum _{i=1}^N\sum _{j=1}^N \delta _{M_{i,j},v_{M}}$ for $v_M\in [0,1]$, and for *I* it is $I_{v_{I}} = \sum _{i=1}^N\sum _{j=1}^N \delta _{I_{i,j},v_I}$ for $v_I\in [-8,8]$. To find the values for the probabilities of these macrostates, independent simulations of the system are run and then the probabilities for the values of each of the macrostates at each iteration given all the different simulation are calculated, with a Monte Carlo approach:

4 $$\begin{aligned} p_C(v_C,t)&= \frac{1}{N_{sim}}\sum _{k=1}^{N_{sim}}\left( \frac{1}{N^2}\sum _{m=1}^{N}\sum _{n=1}^{N} \delta _{v_C,C_{m,n,t,k}}\right) ,\end{aligned}$$

5 $$\begin{aligned} p_M(v_M,t)&= \frac{1}{N_{sim}}\sum _{k=1}^{N_{sim}}\left( \frac{1}{N^2}\sum _{m=1}^{N}\sum _{n=1}^{N} \delta _{v_M,M_{m,n,t,k}}\right) ,\end{aligned}$$

6 $$\begin{aligned} p_I(v_I,t)&= \frac{1}{N_{sim}}\sum _{k=1}^{N_{sim}}\left( \frac{1}{N^2}\sum _{m=1}^{N}\sum _{n=1}^{N} \delta _{v_I,I_{m,n,t,k}}\right) . \end{aligned}$$

Here $N_{sim}$ is the number of independent simulations, and *v* the possible values for the macrostate value of the agents for each of the matrices ($v_C\in [-Cmax,Cmax]$ for *C*, $v_M\in [0,1]$ for *M* and $v_I\in [-8,8]$ for *I*). The Kronecker delta, $\delta$, is used to test for argument equality producing a value of 1 when equal and zero otherwise, $\delta _{x,y} = 1, x=y$ and $\delta _{x,y} = 0, x \ne y$.

Given these probabilities, the entropies of the three matrices which define the state of the agents can be calculated at each simulation time step using the distribution of the values over the grid via:

7 $$\begin{aligned} S_{C,t}&=-\sum _{v_C} p_C(v_C,t)\text {ln}(p_C(v_C,t)) \end{aligned}$$

8 $$\begin{aligned} S_{M,t}&=-\sum _{v_M} p_M(v_M,t)\text {ln}(p_M(v_M,t)) \end{aligned}$$

9 $$\begin{aligned} S_{I,t}&=-\sum _{v_I} p_I(v_I,t)\text {ln}(p_I(v_I,t)). \end{aligned}$$

These political system entropy traces can provide the examiner insight into the overall state of the system for these variables which affect the agents’ political activities.

**Figure 1:**
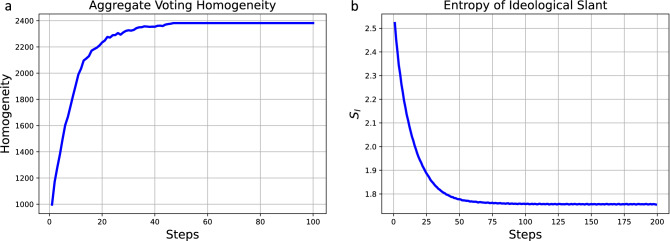
In plot (**a**) the homogeneity of the political choices over the lattice contained in matrix *M* over time is shown. Plot (**b**) shows the entropy for a political component of a system simulation, $S_{I,t}$ with time steps *t*. The entropy decreases until it converges with oscillations. $N_{sim}=1000$ is the number of independent simulations conducted and the average value presented. The dynamics on the agents’ political affiliations (Eq. 1) causes agents to influence each other from their adjacent positions causing them to eventually coalesce. The coalesced state is at a lower entropy than the initial random allocation of ideological positions.

Figure 1 shows 2 plots for the results of running a set of 1000 independent simulations of this model with 100 time steps each. At the initialization each agent receives a sample of their $C_{i,j}$ value from $\mathcal {U}(-Cmax,Cmax)$. In plot (a) the values of the overall homogeneity of the *M* are calculated via,

10 $$\begin{aligned} R(M,t)=\sum _{i=1}^{N}\sum _{j=1}^{N}\left( \sum _{m=i-1}^{i+1}\sum _{n=j-1}^{j+1} \delta _{M_{i,j,t},M_{m,n,t}}\right) , \end{aligned}$$

and in (b) the entropy trace of $S_{I,t}$ is shown. This finds the total number of local ideological leaning agreements per agent on the grid as a measure for how homogeneous the state of the system is. These results show that the dynamics of the system move the state towards one of greater ideological homogeneity and lower the entropy of the overall system. It is seen that with the passage of time the entropy decreases leading to a convergence at a lower value. This is something that is to be expected in an isolated system with rules aiming towards achieving homogeneity^38^, and comes into agreement with previous studies investigating the Schelling model such as in^20,23^. This does not reflect a realistic physical system that would inevitably be expected to respect the second law of thermodynamics as $S_{I,t}$ should be increasing. According to the Gibbs paradox this alludes that the system is not fully defined. Although the model is not designed to be a realistic representation of the actual physical actors it is designed to depict a failure in the modeling approach where a system can be defined which violates a key physical principal, and as a case study a solution to this is presented subsequently.

### Model definitions for the political engagement model with time constrained agent actions

Although there are potentially many variables to consider as candidates to incorporate into the model to address the decreasing entropy trend, such as a monetary variable^23^, it is proposed here that an even more fundamental aspect of the agents can be considered and that is their limited time as a resource. That their time will be shared among other activities outside of the political discourse, and assuming that the amount of time spent in activities is constant; this allows a constraint to be applied. Introducing a constraint between variables provides an intrinsic coupling between variables that can change the state space rather than have associated independent dynamics which are plausibly coupled (such as residential movements and financial expenditure^23,24,34^).

Assuming that each agent can undergo the same number of $n_{total}$ actions at each time step then an agent that engages in a political action will be allowed to perform $n_{total}-1$ non-political actions (activities outside of political discourse). If from $n_{total}-1$ actions each agent on the grid chooses to ’walk’ for example (or eg. ’eat’ or ’read’ etc), then the ’walk’ activity would happen $N^2$ times if chosen once per agent (there are $N^2$ agents). In the case where all the agents choose to exclusively spend all their non-political activities ’walking’ this would be $N^2 \times (n_{total} -1)$ of walking action. This could be a composition of different actions among the agents which leads to the same total aggregate regardless of the actions taken. There is no restriction on the number of different actions an agent can choose from and it is not required to define the size of the set that agents can choose non-political actions from either. This constraint on the number of actions per time unit produces the effect that the more one knows about the $n_{total}$ actions of an agent, the less the uncertainty there is about the total activity set of actions since it subtracts from the unknown actions adding to the known activities. As a consequence, the more agents are engaged in political actions the set of unknown activities becomes smaller. We refer to the agent actions of political discourse as ’political’ actions, and all other activities as ’peripheral’. Due to the unobservable nature of the peripheral activities as defined, the entropy for this component of the system state cannot be found directly. As will be shown this value can be derived using the total system entropy with the entropy of the engagement in political discourse. The benefit of this definition is that no assumptions on the unknown quantities are required.

This simplified model allows for only one political action per time step per agent, and suffices in order to demonstrate its effect. Whether or not an agent will engage in a political action in the next step depends on their current $C_{i,j,t}$ value, and the ideology slant of their neighborhood $I_{i,j,t}$. If the $I_{i,j,t}$ value is non-zero and the $C_{i,j,t}$ value is not on the limit then the agent will engage in political interactions as the agent is not in a homogeneous locality putting agents into the mode of engagement in political discourse. The matrix indicating which of the agents will or will not engage in a political action is ($E_{i,j} \in [0,1]$),

11 $$\begin{aligned} E_{i,j,t}={\left\{ \begin{array}{ll} 1 \,\text {if}\, (I_{i,j,t}\ne 0) \wedge |C_{i,j,t}|\ne C_{max} \\ 0\,\text {if}\, (I_{i,j,t}\ne 0) \wedge (I_{i,j,t}C_{i,j,t}<0) \wedge (|C_{i,j,t}|=C_{max}) \\ 0 \,\text {if} \, I_{i,j,t}=0 \end{array}\right. }. \end{aligned}$$

The non-political actions are considered to be ’peripheral’ actions and in each time step their number for each agent is:

12 $$\begin{aligned} n_{{peripheral}_{i,j,t}} = n_{total}-E_{i,j,t}. \end{aligned}$$

In order to calculate the maximum entropy of this system with no testable information a uniform distribution is applied on the discrete probabilities of the variables which here refer to the peripheral activities. The maximum entropy occurs when the number of political engagements of the agents is 0, and thus $n_{peripheral}=n_{total}$ (maximizing the unknown activities engaged in);

13 $$\begin{aligned} p_{n_{total}} = \frac{1}{n_{total}}. \end{aligned}$$

Therefore the $1/n_{total}$ fraction will get its smallest possible value and the entropy will have its maximum value of:

14 $$\begin{aligned} S_{max} = -\sum _{n=1}^{n_{total}} p_{n_{total}}\text {ln}(p_{n_{total}}). \end{aligned}$$

Since this entropy value does not depend upon the state of the simulation it is constant and represents the maximum possible entropy of the system at all times. As an isolated system without external influence the entropy contained in the variables of this system will have an aggregate summation. This aggregate composed of the entropy of political actions and the entropy of the peripheral actions will be equal to this max entropy value at all times.

The probability of the political engagement by an agent at a time point in a simulation is found from the samples of independent simulations via (where $v_E \in [0,1]$ is the distribution for agents being engaged in political discourse or not),

15 $$\begin{aligned} p_{E}(v_{E},t)=\frac{1}{N_{sim}}\sum _{k=1}^{N_{sim}} \left( \frac{1}{N^2}\sum _{m=1}^{N}\sum _{n=1}^{N}\delta _{v_{E},E_{m,n,t,k}}\right) . \end{aligned}$$

The entropy for these actions of engaging in political discourse is found via:

16 $$\begin{aligned} S_{E,t} = -\sum _{v_E} p_{E}(v_{E},t)\text {ln}(p_{E}(v_{E},t)). \end{aligned}$$

Knowing that the total entropy of the system remains constant during the simulation and that the entropy of the political engagement can be calculated at each time point, the equation for the entropy of the peripheral actions can be produced;

17 $$\begin{aligned} S_{peripheral}=S_{max}-S_{E}, \end{aligned}$$

since $S_{max} = S_{peripheral} + S_{E}$. $S_{E,t}$ varies along the system trajectory depending upon whether the agent is politically engaged or not. $S_{max}$ represents the total entropy of activities an agent is active in, and also takes into account whether there is political engagement by the agent. This allows the system trajectory to be described as a bounded system where the entropy trace of concern is a subset of the complete system whose changes are balanced by the implicitly defined state variables, similarly to physical systems like in^51–53^. As such a model that is compliant with the second law is produced when the variable couplings are accounted for.

## Results

**Figure 2:**
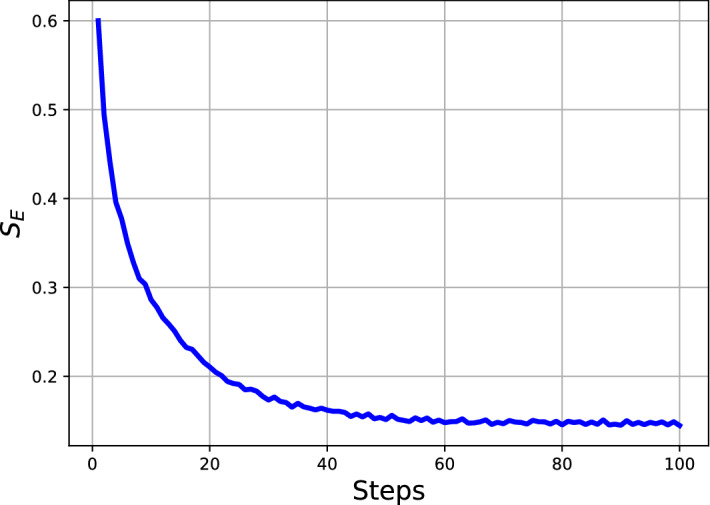
The result of running a set of 200 independent simulations where the entropy of the matrix *E* (Eq. 16) is calculated at each time point and the average across the traces is presented. With the passage of time it can be seen that as the system approaches an increased homogeneity and fewer agents are engaged in political discourse (in a more homogeneous system) this results in lower entropy values.

Figure 2 presents the results of averaging 200 independent simulations of the model of political discourse where at each time point the entropy for the political engagements is estimated using Eq. (11). Each agent begins with a random value for their concealed political affiliation $C_{i,j}$ which for the system is held in Eq. (1) and the dynamics affect these values over time $C_{i,j,t}$. The dynamics drive the system toward greater ideological homogeneity resulting in less political engagements across the agents. From the results in the figure it can be seen how $S_E$ decreases on average since the homogeneity provides fewer expected ideological disparities for which agents then engage in the action of political discourse. $S_E$ is computed via Eq. (16).

The result that there is a decreasing in the entropy for the system should not come as a surprise since the arrangements of the agents upon the grid enter a more ’ordered’ grid state^54^ but it is not common for it to be directly investigated by the community active in social systems research. For instance in^17^ this question is posed directly as “does the second law of thermodynamics apply to social systems or not?” and this figure adds to the evidence that the systems that are not completely defined will not display a respect for the arrow of time.

**Figure 3:**
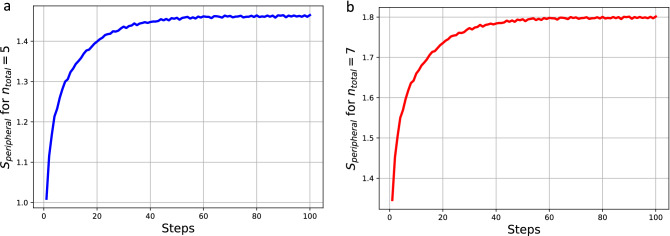
These plots show the results of the average entropy of the peripheral activities (non-politically aligned actions) defined in Eq. (17). On the plot (**a**) $n_{total}=5$ and the plot (**b**) $n_{total}=7$. As the system dynamics drive the agents towards ideological homogeneity the political engagement decreases on average therefore increasing the peripheral agent activities. With the increased average peripheral activity among the agents the entropy of this system component $S_{peripheral}$ increases.

Equation (17) is used to calculate the entropy of the agents in regards to the peripheral actions (those actions that are non-politically aligned), $S_{peripheral}$. The results of taking the average of these values along independent simulation traces is shown in Fig. 3 in two separate plots. Since the simulations can be run with a different number of total actions ($n_{total}$) the plot (a) shows the results of using $n_{total}=5$, and in the plot (b) $n_{total}=7$. In both simulations it can be seen that the entropy of the peripheral actions of the system increases as the dynamics of the political actions are applied. This is due to the homogeneity increase and political engagement decrease driving the increase in peripheral actions. The simulation with $n_{total}=5$ has on average less values than $n_{total}=7$ since there is greater uncertainty for a larger number of actions being chosen by each agent on average. This increase is in contrast to the entropy trace in regards to the political actions which decreases on average with the simulation time steps.

These traces do not emerge from the introduction of exogenous variables into the model definition. There are no assumptions made about the nature of the peripheral activities and even whether they are distinct among each other. This activity set can even include actions such as ’idleness’ since to the system it is another label for which uncertainty exists from the perspective of the observer. When engagement in the observed activity, ’political engagement’, is not present it must be replaced by a label that the observer has no knowledge of (peripheral activity). This has a maximum entropy prior over (the uniform distribution). The entropy will therefore increase when the actions the investigator can observe decreases on average and since it is replaced by unknown activities this adds to the uncertainty.

**Figure 4:**
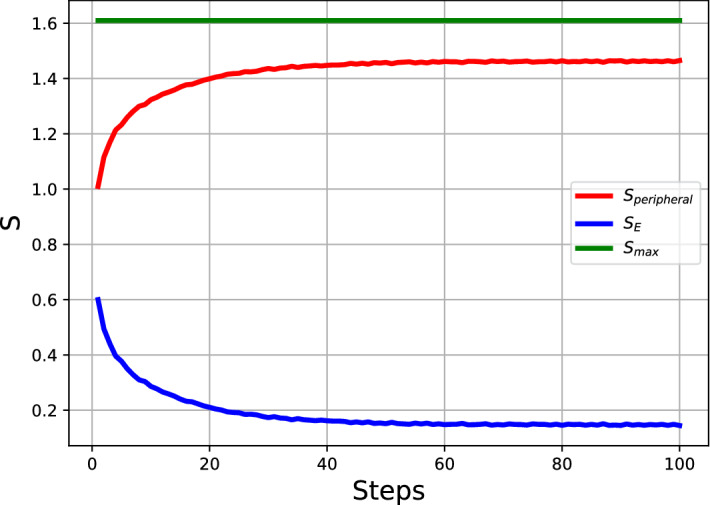
The average of the three entropy traces for the system simulations are shown $S_{max}$, $S_{E}$, and $S_{peripheral}$. The green line shows the $S_{max}$ value is constant which aligns with the theoretical calculation for the entropy of the system, in blue $S_{E}$ the entropy of the political engagement and in red the entropy of non-politically aligned actions $S_{peripheral}$. This simulation has a fixed number of 5 actions ($n_{total}=5$) for the agents to participate in per time point.

Figure 4 shows the results of the trajectory averages along independent simulations of the system where the blue line shows the entropy for the political engagement $S_{E}$, the red line the peripheral action entropy $S_{peripheral}$, and the green line shows the total entropy $S_{max}$. $S_{max}$ is also the aggregate of $S_E$ and $S_{peripheral}$ as worked out theoretically in Eq. (14) and as can be seen from these simulations. The system dynamics producing political homogeneity (Eq. 1) decreases the entropy in respect to political engagement while increasing the entropy in regards to the peripheral activities. These changes are in balance with the system constraint of $S_{max}$ as predicted from Eq. (14). This demonstrates that although the simulation displays a decrease in terms of the political engagement entropy, $S_E$, which is typically the main focus of a social simulation of this sort, it is possible to more fully define the system where the $S_{peripheral}$ compensates for the decrease in entropy. As a result the simulation is then consistent with the second law of thermodynamics. It was not required to introduce a new variable as the constraint is imposed upon already existing variables of the system.

The blue line can be seen as the result of the dynamics of the political discourse on how agent influence each other in the effort to coalesce. This trace has a trend and effect that is analogous to that of the Schelling model. Such a trace (Fig. 2) by itself disregards the intrinsic constraint on the entropy it can express. Having knowledge of this constraint allows us to produce an isolated system formulation as presented in Eq. (14) (shown on the green line). The trace of the model can then be expected to oscillate between known bounds. The difference of the trace and the boundary value produces another trace which as a byproduct is informative to the investigator of the system. Any other entropy defined for this system would necessarily have to be a subset of this trace (red line). The sum of all the non-political engagement entropies produces this red line and therefore the second law of thermodynamics is conserved as we can observe that the overall unknown entropy is increasing.

In context of the goal of understanding political polarization in general this plot brings insight into the conceptual underpinnings of how the agent activities are bounded and distributed. The blue line is the only observable and there is a necessary byproduct which is not observable to the investigator which can increase or decrease based upon the observable blue macrostate value.

**Figure 5:**
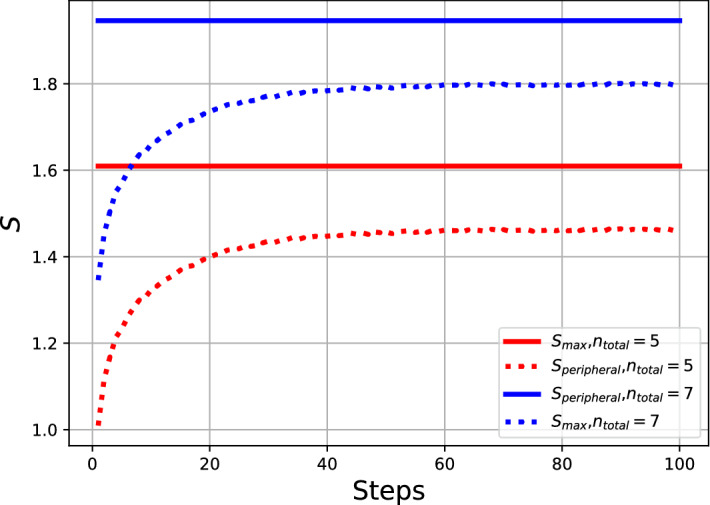
The figure displays the peripheral and maximum entropies of the system when $n_{total}=5$ (red) and $n_{total}=7$ (blue) respectively. The solid lines are the total entropies of the system $S_{max}$ and the dotted lines $S_{peripheral}$. Higher values arise with the increases of the total amount of actions of the agents per time step.

Figure 5 shows the differences between the entropy of the system and for the peripheral actions when setting different values of $n_{total}$. In red are the results from using $n_{total} = 5$ and in blue when $n_{total} = 7$. The solid lines display the value of $S_{max}$ which is constant throughout the simulation and the dotted lines the entropy of the peripheral actions $S_{peripheral}$. It can be seen how the increase in the total number of actions per agent increases the overall system entropy due primarily to the number of peripheral actions which are unknown to the system definition. From results such as this one an investigator can draw conclusions about other components of the system not directly considered knowing the maximum entropy that the system can express and the changes to a particular variable’s relative entropy changes. In the situation where we see the majority of the available uncertainty of the system being contained in a single variable can only result when the uncertainty in the other variables decreases implying some form of homogeneity. The entropy of any non-political engagement of the system is included in this variable $S_{peripheral}$ meaning that for any definition the overall entropy of the uncertain actions of the system is increasing.

**Figure 6:**
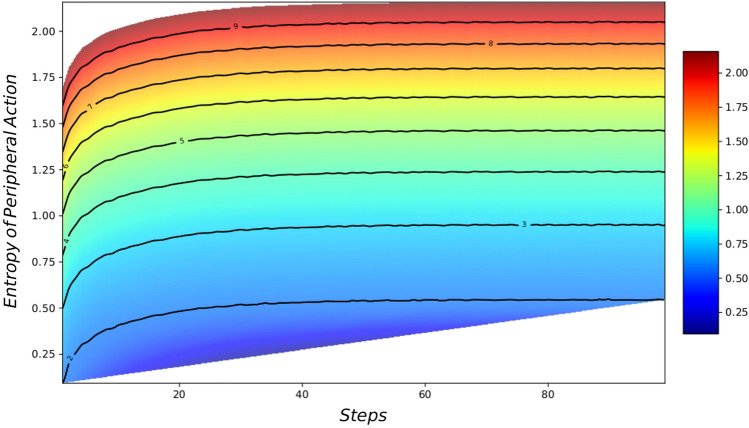
The plot shows the entropies of the peripheral actions ($S_{peripheral}$) of the agents for the different number of total available actions (Dark Blue has $n_{total}=2$ actions, Red has $n_{total}=10$ actions). It can be seen that the entropy takes higher values as the total amount of the actions of the agents increases. The general trend is that the agents produce more ideological homogeneity over time increasing the uncertainty in the peripheral activities of the agents.

Figure6 displays the results from a set of simulations which are run with different values of $n_{total}$. Starting from the bottom $n_{total}=2$ and continuing to the highest line where $n_{total}=9$. This demonstrates that as the simulations progress and the opinions across agents coalesce the average number of peripheral actions increases as less engagement in political discourse is relevant to agents within ideologically homogeneous localities. The entropy of the peripheral actions increases according to Eq. (17) over time steps and for larger values of $n_{total}$. It is observed that the constraint on the number of actions an agent can partake in per unit of time increases or decreases the overall entropy without altering the general trend. This highlights a key aspect that the political engagement acts to reduce the amount of entropy as this action is a known observable, and thus reducing the uncertainty from the perspective of the observer.

## Discussion

The goal of this manuscript has been to investigate the dynamics of political polarization. A key issue is that it is possible to define a set of dynamics which do not respect the un-moving principles of thermodynamics which affect all levels of complex systems. Dynamics can be defined in regards to isolated aspects of the system that ignore the balancing effects. The model described in this work demonstrates such an issue, and how it can be alleviated via the definition of an isolated system.

A simple lattice model of political discourse is proposed in which agents do not change position while influencing each other, and agents only engage in political discourse when they reside in a locality that is not ideologically homogeneous. The methodological formulations put forward show how the aggregate entropy for the agents’ activities can be defined where the entropy at the simulation steps can be sampled. These entropy traces show a decrease in entropy as the average agent homogeneity across the grid increases. A constraint upon the agents is imposed where the total number of actions an agent can engage in per time unit is a constant. Outside of political engagements, a set of non-political engagements exists where this set is a range of unknown activities upon which no assumptions are made. It is shown how the full entropy of the system can be calculated theoretically and this constant value is observed through the simulation study results. This maximum entropy value is constant in regards to the entropy of the aggregate of the politically and non-politically aligned actions.

The results of the simulation of this hypothetical system show that the theoretical treatment of the model of political polarization conforms to the constraints imposed. The key takeaways are that it is possible to define a model of political polarization that is treated as an isolated system in respect to the entropy traces. The entropy trace for political engagement decreases as the homogeneity along political ideology increases. The variables which account for this entropic balancing are not introduced through external factors but via an intrinsic constraint imposed upon the total activity set of agents over time (as this will have to be bounded realistically). As the entropy trace in regards to political activities decreases due to the achieved homogeneity the set of non-politically aligned actions increases driving its entropy trace up in exactly the same amount as the other decreases. These counterbalancing changes produces a constant value equal to the theoretically formulated maximum entropy of the isolated system. It is therefore possible to view political polarization as a component of the total entropy which a system can display that has theoretical bounds. Future work entails the investigation of how the driving force behind the system must have energetic constraints as well. By using the approach developed in this work the rest of the thermodynamic variables, including the energy, can be defined based on the entropy and in the end provide a better understanding of the driving force, and what would the meaning of energy be in such a system.

## Data Availability

All data generated or analysed during this study are included in this published article.

## References

[1] Stewart JQ (1950). The development of social physics. Am. J. Phys..

[2] Stewart JQ (1948). Concerning social physics. Sci. Am..

[3] Jusup M (2022). Social physics. Phys. Rep..

[4] Perc M (2017). Statistical physics of human cooperation. Phys. Rep..

[5] Castellano C, Fortunato S, Loreto V (2009). Statistical physics of social dynamics. Rev. Mod. Phys..

[6] D’Orsogna MR, Perc M (2015). Statistical physics of crime: A review. Phys. Life Rev..

[7] Góis AR, Santos FP, Pacheco JM, Santos FC (2019). Reward and punishment in climate change dilemmas. Sci. Rep..

[8] Garibay I, Mantzaris AV, Rajabi A, Taylor CE (2019). Polarization in social media assists influencers to become more influential: Analysis and two inoculation strategies. Sci. Rep..

[9] Valensise CM (2021). Entropy and complexity unveil the landscape of memes evolution. Sci. Rep..

[10] Porter TM (1981). A statistical survey of gases: Maxwell’s social physics. Hist. Stud. Phys. Sci..

[11] Boltzmann, L. The second law of thermodynamics. in *Theoretical Physics and Philosophical Problems*. 13–32 (Springer, 1974).

[12] Ozawa, H., Ohmura, A., Lorenz, R. D. & Pujol, T. The second law of thermodynamics and the global climate system: A review of the maximum entropy production principle. *Rev. Geophys.* **41** (2003).

[13] Kleidon, A., Malhi, Y. & Cox, P. M. *Maximum Entropy Production in Environmental and Ecological Systems* (2010). 10.1098/rstb.2010.0018 PMC2871911 20368247

[14] Chapman EJ, Childers DL, Vallino JJ (2016). How the second law of thermodynamics has informed ecosystem ecology through its history. BioScience.

[15] Koutsoyiannis D, Sargentis G (2021). Entropy and wealth. Entropy.

[16] Jakimowicz A (2020). The role of entropy in the development of economics. Entropy.

[17] Mavrofides T, Kameas A, Papageorgiou D, Los A (2011). On the entropy of social systems: A revision of the concepts of entropy and energy in the social context. Syst. Res. Behav. Sci..

[18] Bailey KD (2009). Entropy systems theory. Syst. Sci. Cybern. (Eolss Publishers, Oxford, UK).

[19] Styer DF (2000). Insight into entropy. Am. J. Phys..

[20] Mantzaris AV, Marich JA, Halfman TW (2018). Examining the Schelling model simulation through an estimation of its entropy. Entropy.

[21] Schelling TC (1971). Dynamic models of segregation. J. Math. Sociol..

[22] Schelling TC (2006). Micromotives and Macrobehavior.

[23] Mantzaris AV (2020). Incorporating a monetary variable into the Schelling model addresses the issue of a decreasing entropy trace. Sci. Rep..

[24] Hatna E, Benenson I (2012). The Schelling model of ethnic residential dynamics: Beyond the integrated-segregated dichotomy of patterns. J. Artif. Soc. Soc. Simul..

[25] Amin TG, Jeppsson F, Haglund J, Strömdahl H (2012). Arrow of time: Metaphorical construals of entropy and the second law of thermodynamics. Sci. Educ..

[26] Black. *Thermodynamics SI Version* (Addison-Wesley Longman, Incorporated 1992).

[27] Gibbs, J. W. *Elementary Principles in Statistical Mechanics: Developed with Especial Reference to the Rational Foundations of Thermodynamics* (C. Scribner’s Sons, 1902).

[28] Jaynes, E. T. The Gibbs paradox. in *Maximum Entropy and Bayesian Methods*. 1–21 (Springer, 1992).

[29] Avetisov V, Gorsky A, Maslov S, Nechaev S, Valba O (2018). Phase transitions in social networks inspired by the Schelling model. Phys. Rev. E.

[30] Stauffer D, Solomon S (2007). Ising, Schelling and self-organising segregation. Eur. Phys. J. B.

[31] Müller K, Schulze C, Stauffer D (2008). Inhomogeneous and self-organized temperature in Schelling-Ising model. Int. J. Mod. Phys. C.

[32] Cheng X, Liang X, Guo Z (2011). Entransy decrease principle of heat transfer in an isolated system. Chin. Sci. Bull..

[33] Sears, F., Salinger, G. & Lee, J. *Thermodynamics, Kinetic Theory, and Statistical Thermodynamics. Addison-Wesley Principles of Physics Series* (Addison-Wesley Publishing Company, 1975).

[34] Hatna, E. & Benenson, I. Combining segregation and integration: Schelling model dynamics for heterogeneous population. arXiv preprint arXiv:1406.5215 (2014).

[35] Dixit AK, Weibull JW (2007). Political polarization. Proc. Natl. Acad. Sci..

[36] Gentzkow, M. Polarization in 2016. in *Toulouse Network for Information Technology Whitepaper*. 1–23 (2016).

[37] Janoff-Bulman R, Carnes NC (2016). Social justice and social order: Binding moralities across the political spectrum. PloS one.

[38] Domenikos, G.-R. & Mantzaris, A. V. A model simulation of political segmentation through an estimation of the entropy. *J. Stat. Mech. Theory Exp.*10.1088/1742-5468/ac8800 (2022).

[39] Baldassarri D, Bearman P (2007). Dynamics of political polarization. Am. Sociol. Rev..

[40] Wilson, J. Political discourse. in *The Handbook of Discourse Analysis*. 398–415 (2005).

[41] Delanty G (2002). Two conceptions of cultural citizenship: A review of recent literature on culture and citizenship. Glob. Rev. Ethnopolitics.

[42] Van Dijk TA (2006). Discourse and manipulation. Discourse Soc..

[43] Van Dijk TA (2006). Ideology and discourse analysis. J. Political Ideol..

[44] Molinero X, Riquelme F (2021). Influence decision models: From cooperative game theory to social network analysis. Comput. Sci. Rev..

[45] Sweet T, Adhikari S (2020). A latent space network model for social influence. Psychometrika.

[46] Bekenstein JD (2003). Information in the holographic universe. Sci. Am..

[47] Susskind L (1995). The world as a hologram. J. Math. Phys..

[48] Fein E (1970). Demography and thermodynamics. Am. J. Phys..

[49] Axelrod, R., Daymude, J. J. & Forrest, S. Preventing extreme polarization of political attitudes. *Proc. Natl. Acad. Sci.* **118** (2021). 10.1073/pnas.2102139118 PMC8685667 34876506

[50] Ising E (1925). Contribution to the theory of ferromagnetism. Z. Phys..

[51] Svidzinsky A, Kim M, Agarwal G, Scully MO (2018). Canonical ensemble ground state and correlation entropy of Bose-Einstein condensate. New J. Phys..

[52] Domenikos, G.-R., Rogdakis, E. & Koronaki, I. Studying the superfluid transformation in helium 4 through the partition function and entropic behavior. in *ASME International Mechanical Engineering Congress and Exposition* (American Society of Mechanical Engineers, 2021).

[53] Domenikos, G.-R., Rogdakis, E. & Koronaki, I. Thermodynamic correlation of the entropy of Bose-Einstein condensation transition to the lambda points of superfluids. *J. Energy Resour. Technol.* 1–10 (2022).

[54] Wright P (1970). Entropy and disorder. Contemp. Phys..

